# *PALB2 *variants in hereditary and unselected Finnish Prostate cancer cases

**DOI:** 10.1186/1477-5751-8-12

**Published:** 2009-12-15

**Authors:** Sanna Pakkanen, Tiina Wahlfors, Sanna Siltanen, Mimmi Patrikainen, Mika P Matikainen, Teuvo LJ Tammela, Johanna Schleutker

**Affiliations:** 1 Laboratory of Cancer Genetics, Institute of Medical Technology, University of Tampere and Tampere University Hospital, Tampere, Finland; 2 Department of Urology, Tampere University Hospital and Medical School, University of Tampere, Tampere, Finland

## Abstract

**Background:**

*PALB2 *1592delT mutation is associated with increased breast cancer and suggestive prostate cancer (PRCA) risk in Finland. In this study we wanted to assess if any other *PALB2 *variants associate to increased PRCA risk and clinically describe patients with formerly found *PALB2 *1592delT mutation.

**Methods:**

Finnish families with two or more PRCA cases (n = 178) and unselected cases (n = 285) with complete clinical data were initially screened for variants in the coding region and splice sites of *PALB2*. Potentially interesting variants were verified in additional set of unselected cases (n = 463).

**Results:**

From our clinically defined sample set we identified total of six variants in *PALB2*. No novel variants among Finnish PRCA cases were found. Clinical characteristics of the variant carriers, including the previously described family carrying *PALB2 *1592delT, revealed a trend towards aggressive disease, which also applied to a few non-familial cases. Hypersensitivity to mitomycin C (MMC) of lymphoblasts from individuals from the family with 1592delT revealed haploinsufficiency among carriers with altered genotype.

**Conclusions:**

Though any of the detected *PALB2 *variants do not associate to PRCA in population level in Finland it cannot be ruled out that some of these variants contribute to cancer susceptibility at individual level.

## Introduction

Prostate cancer (PRCA) is the most frequently diagnosed cancer among men in the Western world [1]. The incidence of PRCA has been increasing throughout the last decade, partly due to the earlier diagnosis provided by the prostate specific antigen (PSA) test, introduced in the 1990s. In Finland, the highest incidence of 115/100 000 was observed in 2005. In 2007 there were 4188 newly-diagnosed PRCA cases with the age-adjusted incidence rate of 85.9/100,000 [2]. Similar high increase and now decline have been observed in most Western industrialized countries [3].

Extensive efforts have been made to reveal the genetics behind PRCA susceptibility [4]. Early linkage studies and more recent genome-wide association analyses have revealed multiple susceptibility loci for PRCA [5-7]. However, both causal variants and genes of the associated loci in are still not known and the identified PRCA risk variants only account for a fraction of the overall genetic variance for PRCA risk [8-10].

DNA repair pathway genes have an undisputed role in cancer progression and inherited mutations in them have been strongly associated with different cancers. *BRCA1 *and *BRCA2 *are both tumor suppressor genes involved in DNA repair and mutations in these genes predominantly predispose carriers to breast and ovarian cancers [11]. Potential links to PRCA have been studied for both of these genes and mutations in *BRCA2 *lead to an increased risk for the disease in multiple studies [12,13]. In an Icelandic study, mutations in *BRCA2 *(999del5, Icelandic founder mutation) are associated with a poorly differentiated, advanced type of PRCA [14]. These results are also consistent with results from a recent study in the UK, where a significantly higher Gleason score was observed among *BRCA2 *mutation carriers than with non-carriers. These findings suggest that *BRCA2 *is a high-risk PRCA susceptibility gene and mutational analysis could be used as a prognostic marker for aggressive PRCA [15]. However, Finnish *BRCA1/2 *founder mutations do not associate with PRCA predisposition among Finnish PRCA patients [16]. This does not exclude the possibility of these genes to be involved in PRCA susceptibility but no studies have been published where the whole genes would have been sequenced in large data sets. Nevertheless an increased risk for PRCA in Finnish breast cancer families carrying *BRCA2 *mutations has been observed [17]. Moreover, mutations of *CHEK2*, a gene acting in the same DNA repair route as *BRCA1/2*, have been shown to have a significant role in PRCA susceptibility in Finland [18].

PALB2 is a BRCA2 binding protein and the BRCA2-PALB2 interaction is essential for BRCA2-mediated DNA repair [19]. Recently it was shown that proper PALB2 function is necessary for the homologous recombination repair via interaction with BRCA1, revealing that PALB2 is actually a linker between BRCA1 and BRCA2 [20]. In the Finnish study by Erkko and colleagues a novel *PALB2 *founder mutation (c.1592delT) was identified among Finnish breast cancer families (OR 11.3, CI 1.8-57.8) increasing the risk to breast cancer 4-fold [21]. In the same study the c.1592delT mutation was observed also from a patient with familial PRCA (1/164) but none of 475 unselected cases were carriers of the mutation. No statistically significant association with PRCA and c.1592delT was detected and no other variants were screened for in PRCA patients. Here, to investigate the role of possible other *PALB2 *variants, we screened all *PALB2 *exons(exons 1-13) in a cohort of 938 Finnish PRCA patients including both familial and unselected cases.

## Material and methods

### Patients and controls

A population-based cohort was collected from 1999 to 2005 from patients diagnosed with PRCA in the Pirkanmaa Hospital District that serves a population of a half a million inhabitants. The mean age at diagnosis for the 760 unselected patients was 62 (range 43-77), The median primary PSA and Gleason score were 8.9 and 6.4, respectively. The controls (n = 760) consisted of DNA samples from anonymous male blood donors obtained from the Finnish Red Cross.

A detailed description of the collection of the PRCA families has been previously described elsewhere [22]. In this study, we analysed 178 families (youngest affected male from each family) with two or more affected first-degree relatives. The average number of affected family members was three. Familial cancer data was attained from the Finnish Cancer Registry and detailed clinical information, including Gleason score, WHO grade, PSA at the time of diagnosis, TNM stage and primary treatment from hospital records.

To examine the association between the variants and the disease aggressiveness a subgroup of 380 with clinically aggressive disease was analyzed. Consequently clinically aggressive disease patients were selected according to Gleason score over seven and an age at diagnosis less than 61 years.

### Mutation analysis of PALB2

All familial samples had been previously screened for the 11 Finnish *BRCA1 *and seven *BRCA2 *founder mutations [16]. DNA samples from each family (n = 178), 285 unselected cases and 470 control samples were initially used for direct sequencing of the entire coding region and splice sites of *PALB2*. Unselected cases for screening the whole gene were early onset cases with Gleason score over seven. Additional analyses were carried out on four variants that showed a trend for association (*PALB2 *c.1592delT, 1674A>G, 2993G>A and 3300T>G). Unselected cases for additional analysis included 95 early onsets, aggressive cases and 368 non-aggressive cases with average age at diagnosis 67 years (range 63-77). Control samples in both primary and additional analysis were anonymous male blood donors from the Finnish Red Cross.

Mutation detection was performed through resequencing using the ABI PRISM BigDye Terminator Cycle Sequencing Ready Reaction kit with the ABI 3130xl sequencer (Applied Biosystems). Primers and the naming of sequence variants were produced to correspond to the GenBank reference sequences for *PALB2 *(NM_024675.3). Primer sequences are available on request. Variants were identified using Sequencher software 4.7 (Gene Codes Corporation, Ann Arbor, Mi). Loss of heterozygosity (LOH) analysis was carried out on two available tumor samples from the family with the segregating *PALB2 *1592delT. Selected sections of formalin fixed paraffin embedded tumors were obtained according to pathologist declaration. After tumor deparaffinization, DNA was extracted using a standard proteinase K protocol. Sequence from the tumor samples was compared to the patients' DNA sample from their peripheral blood.

### DNA damage response in PALB2 deficient cell line

Peripheral blood leucocytes from two cancerous individuals carrying *PALB2 *1592delT mutation were immortalized with Epstein-Barr virus (EBV) and sensitized to DNA crosslinking agent mitomycin C (MMC). Cells were treated with 0 nM, 1 nM, 5 nM, 10 nM and 50 nM MMC for 96 h and their viability was assayed by CellTiter-Blue^® ^Cell Viability Assay (Promega Corporation, Madison, WI, USA).

### Statistical analyses

To validate significance of variants, the odds ratios and 95% confidence intervals were calculated and association analyses were made using the Fisher's exact test. Analyses were done using SPSS 12.0 statistical software. Pairwise linkage disequilibrium between three most frequently detected variants (1674A>G, 2993G>A and 3300T>G) in *PALB2 *were analysed with Haploview 4.1. To define the haplotypes of cases familial and unselected cases were combined.

## Results and Discussion

### Mutation analysis

**Figure 1:**
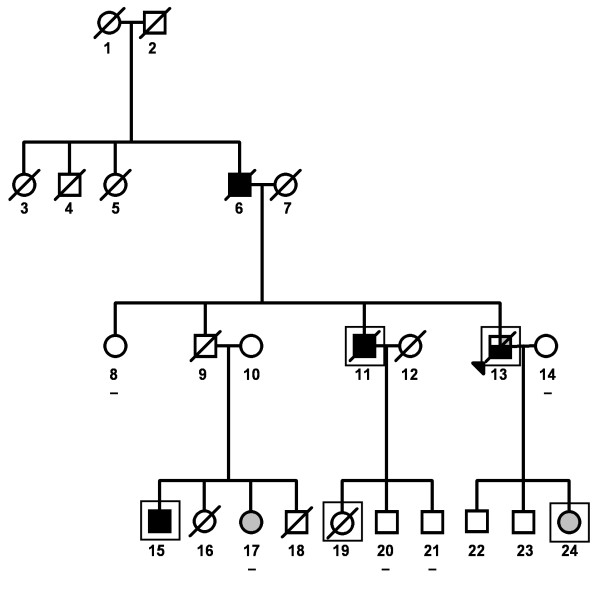
**Family 310 segregating the *PALB *mutation**. Minus-symbol signifies a person that has been screened for the *PALB2 *1592delT mutation, but found negative. Clinical information on this family is found in Table 2. Index person is marked with a black triangle, black square denotes persons with prostate cancer, grey circle indicates breast cancer cases and black square with white and grey corners signifies patient with prostate, stomach and skin cancers.

Prompted by the previous observation that the Finnish truncating founder mutation, *PALB2 *c.1592delT, was identified segregating in one family with four PRCA cases [21], we wanted to study the contribution of other possible *PALB2 *mutations. Probands from 178 Finnish HPC (hereditary prostate cancer) families and 285 unselected cases were screened for the entire coding region of *PALB2*. Total of six variants were detected in the coding regions and the exon-intron boundaries of *PALB2 *(Table 1). All the variants have been previously described and only the truncating mutation (c.1592delT) has been shown to have functional consequences on DNA damage response. Association of four detected variants (c.1592delT, 1674A>G, 2993G>A and 3300T>G) were assessed in more detail in a larger set of unselected cases and controls. The truncating c.1592delT mutation was detected in two sporadic cases in addition to the previously described family 310 and in one control sample [21]. Interestingly, family 310 has now been found to have also stomach and skin cancer, in addition to earlier described breast cancer, as indicated in Figure 1 and Table 2. Three of the detected variants, 1674A>G in exon 4, 2993G>A in exon 9 and 3300T>G in exon 12, co-existed in six patients out of 178 familial samples (OR 1.52 CI 95% 0.6-3.9), 20/748 unselected cases (OR 1.20 CI 95% 0.6-2.3) and in 17/760 of the controls. To assess question whether these three variants have a stronger joint effect that would strengthen the trend seen with singletons a haplotype analysis was performed. The strongest linkage was between 1674A>G and 2993G>A (D'= 0.975; r^2 ^= 0.14, LOD 34.7) when 1886 case chromosomes and 1714 control chromosomes were analysed. Although these two variants appeared to have a frequency in PRCA cases that is higher than the Finnish population control frequency, their combined haplotype gave no improved results, i.e. none of the haplotype combinations showed significant association with the disease. Variant 2993G>A is now reported for the first time in the Finnish population since it did not come up in the study of Finnish breast cancer patients [21]. As reported by Rahman and colleagues [23] the 2993G>A variant is possibly damaging to protein function, based on SIFT (sorting intolerant from tolerant) analysis.

**Table 1:** Observed *PALB2 *variants among the Finnish familial and unselected prostate cancer cases.

Exon/Intron	Nt change	Amino acid Change	Carrier Frequency (OR; 95% CI)		
			
			Familial	Unselected	Controls
***PALB2***					
Ex 4	1592delT	Leu531>Fs>Stop	1/178 (0.6%) (4.3; 0.3-68.8)	2/748 (0.3%) (2.0; 0.2-22.2)	1/760 (0.2%)
Ex 4	1674A>G	Gln559Agr	36/178 (20.2%) (1.2; 0.8-1.8)	153/748(20.5%) (1.2; 0.9-1.5)	134/760(18.0%)
Ex 8	2794G>A	Val932 Met	4/178 (2.2%) (1.0; 0.3-3.4)	7/285(2.5%) (1.2; 0.4-3.1)	10/470 (2.1%)
Ex 9	2993G>A	Gly998Glu	8/178 (4.5%) (1.6; 0.7-3.5)	35/748 (4.9%) (1.6; 0.9-2.8)	22/760 (2.9%)
Ex 12	3300T>G	Thr1100Thr	6/178 (3.4%) (0.8; 0.3-2.0)	30/748 (3.9%) (1.0: 0.6-1.7)	30/760 (3.9%)
5'UTR	G>A	-	8/178 (4.5%) (1.8; 0.7-4.4)	14/285 (4.9%) (2.0; 0.9-4.3)	12/470 (2.6%)

**Table 2:** Clinical characteristics of family 310. The patient number refers to the number in the pedigree in Figure 1.

Patient	Cancer	Histology	Age at diagnosis	Reason for diagnosis	First treatment	Primary PSA value	WHO grading	Gleason grading	TNM grading	IHC
6	Prostate	Adenocarcinoma	83	NA	NA	NA	NA	NA	NA	
11	Prostate	Adenocarcinoma	68	Symptoms	Orchiectomy	NA	III	Na	T3NXMX	
13	Stomach	Adenocarcinoma	70	Symptoms	Gastrectomy	NA	NA	Na	T4N0 M0	
13	Skin	Carcinoma epidermoides	75	NA	Resection	NA	NA	Na	NA	
13	Prostate	Adenocarcinoma	76	Elevated PSA	Orchiectomy	47	III	2+3 = 5	T2NXMX	
15	Prostate	Adenocarcinoma	69	Elevated PSA	Brakytherapy	7.7	I	2+3 = 5	T1cNXMX	
17	Breast	Ductal carcinoma	52	Screening mammography	Mammary resection	NA	I	Na	T1N0 M0	ER+, PR+
24	Breast	Ductal carcinoma	49	Mammography	Mammary resection	NA	I	Na	T1N0 M0	NA

IHC = immunohistochemistry, NA = not available

We found that the 2993G>A variant exhibits a borderline significant odds ratios in a subgroup of 380 unselected cases with a Gleason score over seven and an age at diagnosis less than 61 years (20/380; OR 1.9; 95% CI 1.0-3.5). Edwards and colleagues [24] have presented similar age related observation with *BRCA2 *mutations in prostate cancer. They reported protein truncating mutations in *BRCA2 *to associate with PRCA diagnosed at or before age of 55. Our subgroup analysis also revealed that the variant 1674A>G has an OR of 1.4 (86/380; 95% CI 1.0-1.9) in a group of 368 patients with an average age at diagnosis of 67 years. However, the 1674A>G variant is commonly found in the Finnish population and well tolerated according to SIFT analysis, indicating no clear contribution to cancer formation. Similarly, the Val932 Met (2794G>A) change in exon 8 is well tolerated and has no effect on protein function [23].

In order to obtain information about loss of heterozygosity in the tumors from c.1592delT mutation positive patients, two available paraffin-embedded tumors were analysed. Repeated PCR-analysis did not reveal existence of LOH, which argues against the role as tumor suppressor gene. However the sample size is very small and therefore more tumor samples from *PALB2*-associated tumors needs to be characterized to be able to reliable assess PALB2 function in tumor formation. To date there is only one report indicating LOH in *PALB2 *related tumors. Unfortunately also this study lacks a statistical power to make an appropriate conclusion about the role of PALB2 in tumorigenesis [25].

### DNA damage response in PALB2 deficient cell line

**Figure 2:**
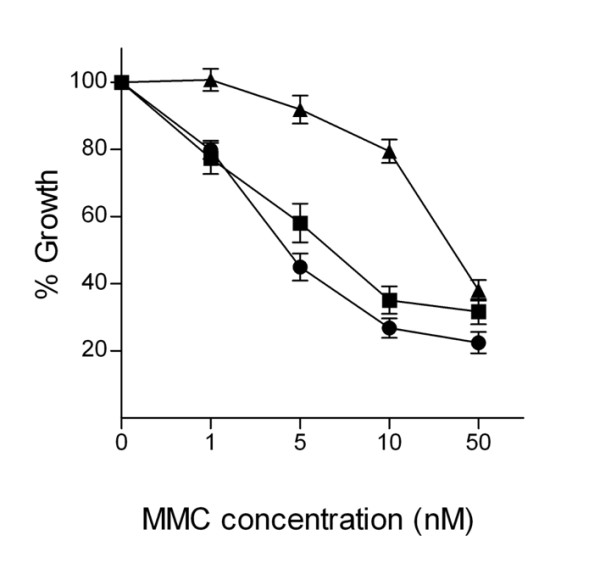
**Mitomycin C growth inhibition in lymphoblastoid cell lines from the 310 family**. Black circle and square depicts cell lines derived from affected individuals with *PALB2 *1592delT mutation. Black triangle signs for control sample from healthy individual.

Previously, MMC sensitivity test to c.1592delT mutation has been done in a reporter cell line [21]. To test whether the previously described *PALB2 *c.1592delT mutation has functional consequences also in a heterozygous form, as present in all our patients, we predisposed two lymphoblast cell lines with c.1592delT and a wild type carrying control cell line to DNA cross linking agent MMC (Figure 2). c.1592delT mutation carriers were patients (15 and 24) from family 310 earlier described by Erkko et al. [21]. In both cell lines MMC-induced growth inhibition was observed suggesting PALB2 haploinsufficiency. It is also possible that dominant-negative effect is affecting in tumor formation in these patients.

Geographical and ethnical differences in the PRCA susceptibility alleles have been previously reported in the *CHEK2 *gene that acts in the same pathway as *PALB2*, and therefore, variation in the *PALB2 *mutation spectrum in different populations would not be surprising. In the present study and in the study by Erkko et al. [21], the number of mutations found in *PALB2 *is relatively small among both Finnish breast and PRCA patients, with nine and six variants detected, respectively, compared to the British breast cancer family study by Rahman and colleagues [23] where a total of 50 variants and five frameshift mutations were reported. Recently, Tischkowitz and colleagues [26] reported a study of 95 US PRCA patients from Michigan, all diagnosed at < 55 years of age. Eleven variants in *PALB2 *were found, but none of them were truncating and no association with the disease was observed. When comparing our patients to those very early-onset patients studied by Tischkowitz and colleagues [26] there is a clear difference in the mean age of onset between the two datasets. On the other hand, no variants were found in the 14 Ashkenazi-Jewish and 21 French-Canadian PRCA cases with family history of cancer [27]. In Finland, the smaller number of variation found likely reflects the known genetic homogeneity and founder effect of the population [28].

Our findings indicate that no other deleterious *PALB2 *variants, except 1592delT mutation, contribute even marginally to PRCA risk in Finland. However, it remains possible that other genes from the BRCA1-PALB2-BRCA2 pathway have PRCA-predisposing alleles

## Competing interests

The authors declare that they have no competing interests.

## Authors' contributions

SP gathered the clinical data and aggregated the family structures of PRCA families and drafted the manuscript. TW and SS genotyped the cases and the controls. TW did the statistical calculations, contributed to study design and coordination and revised the manuscript. MP performed the MMC sensitivity test. MM and TT are the clinical contributors. JS participated in study design, interpreted the results and critically revised the manuscript. All the authors have read and approved the final manuscript.
